# Epigenetic regulation of TGF-β-induced EMT by JMJD3/KDM6B histone H3K27 demethylase

**DOI:** 10.1038/s41389-021-00307-0

**Published:** 2021-02-26

**Authors:** Su-Hyun Lee, Okhwa Kim, Hyo-Jin Kim, Cheol Hwangbo, Jeong-Hyung Lee

**Affiliations:** 1 Department of Biochemistry, Kangwon National University, Chuncheon, Gangwon-do 24341 Republic of Korea; 2 Kangwon Institute of Inclusive Technology (KIIT), Kangwon National University, Chuncheon, Gangwon-do 24341 Republic of Korea; 3 Division of Applied Life Science (BK21), PMBBRC and Research Institute of Life Sciences, Gyeongsang National University, Jinju, Gyeongsangnam-do 52828 Republic of Korea

**Keywords:** Breast cancer, Cancer microenvironment

## Abstract

Transforming growth factor-β (TGF-β) signaling pathways are well-recognized for their role in proliferation and epithelial–mesenchymal transition (EMT) of cancer cells, but much less is understood about their contribution to interactions with other signaling events. Recent studies have indicated that crosstalk between TGF-β and Ras signaling makes a contribution to TGF-β-mediated EMT. Here, we demonstrate that Jumonji domain containing-3 (JMJD3 also called KDM6B) promotes TGF-β-mediated Smad activation and EMT in Ras-activated lung cancer cells. JMJD3 in lung cancer patients was significantly increased and JMJD3 expression in lung tumor tissues was correlated with expression of K-Ras or H-Ras in particular, and its expression was regulated by Ras activity in lung cancer cells. JMJD3 promotes TGF-β-induced Smad activation and EMT in Ras-activated lung cancer cells through the induction of syntenin, a protein that regulates TGF-β receptor activation upon ligand binding. Tissue array and ChIP analysis revealed that JMJD3 epigenetically induces syntenin expression by directly regulating H3K27 methylation levels. Mechanical exploration identified a physical and functional association of JMJD3 with syntenin presiding over the TGF-β in Ras-activated lung cancer cells. Taken together, these findings provide new insight into the mechanisms by which JMJD3 promotes syntenin expression resulting in oncogenic Ras cooperation with TGF-β to promote EMT.

## Introduction

Transforming growth factor-β (TGF-β) is one of the main growth factors involved in driving EMT. Increased TGF-β expression is found in human breast cancers, particularly in invasive human breast cancers and high expression correlated with tumor progression, metastasis, increased recurrence, and poor prognosis^1^. TGF-β1 signals by activating TGF-β type I receptor (TβRI) and TGF-β type II receptor (TβRII) heteromeric complexes, and activated TβRI phosphorylates receptor-associated Smad2 and Smad3. Phosphorylated Smad2 and Smad3 form stable complexes with co-Smad, Smad4, and these then translocate to the nucleus, where they regulate transcription of target genes^2–7^. In a process that depends on the cellular context, TGF-β can induce EMT in cooperation with oncogenic Ras^8–10^. More than 60 percent of human primary breast cancers, exhibit increased oncogenic Ras protein expression, and thus Ras expression was suggested to be a marker of tumor aggressiveness in breast cancer^11^. Through synergistic interactions that support EMT in the presence of TGF-β, it has been shown that oncogenic Ras acts with TGF-β to facilitate cell migration in vitro and tumor invasion and metastasis in vivo^12–15^. The mechanism by which oncogenic Ras contributes to EMT in concert with TGF-β is not yet understood.

In conjunction with DNA methylation, epigenetic mechanisms largely manifest through histone modifications, which make the gene promoter either accessible or inaccessible to transcription factors depending on the nature and location of modifications^16,17^. Histone H3 lysine trimethylation (H3K27me3) on gene promoters mediated by polycomb proteins is one of these essential histone modifications and silences gene transcription^18–21^. The Jumonji domain containing-3 (JMJD3), also known as lysine (K)-specific demethylase 6B (KDM6B), is a member of the Fe(II)- and α-ketoglutarate-dependent demethylases that activate gene expression by removing H3K27me3 marks on gene promoters^22–24^. JMJD3 catalyzes the transition from a repressive to an active chromatin conformation of H3K27me3^24–26^. JMJD3 has been shown to be involved in tumor progression via regulation of cell proliferation, migration, and senescence^26–29^, and its expression is significantly increased in human prostate cancer cells, gliomas, and renal cancer cells compared to adjacent normal tissue^30,31^. High level of JMJD3 induces the expression of mesenchymal genes such as Snail and Slug, which promote TGF-β-induced EMT and tumor metastasis^32–34^.

We have recently shown that syntenin, which contains a tandem PDZ domain, has increased expression in lung cancer cells, complexes directly with TβRI at the plasma membrane, and regulates TGF-β1-induced Smad activation and EMT transition by inhibiting TβRI internalization and degradation^13^. In this study, we present data showing that JMJD3 is a key regulator for syntenin expression, which in turn, promotes the TGF-β1-induced Smad signals and EMT pathways. JMJD3 was found to be highly expressed in human lung cancer cells compared to normal tissues, and JMJD3 expression is regulated by Ras in particular. Furthermore, JMJD3 expression in lung tissues was inversely correlated with patient survival. JMJD3 binds to the promoter of syntenin and demethylates H3K27me3 and has a positive correlation with syntenin expression based on Ras activity in lung cancer cells and tissues. We further demonstrate that JMJD3 regulates TGF-β-induced EMT through a syntenin-TβR1 complex, subsequent activation of Smad2/3, cancer cell migration, and invasion. These functional effects of JMJD3 were exerted through control of syntenin transcriptional expression via H3K27me3. Our findings provide a novel mechanistic role for JMJD3 in linking the crosstalk between TGF-β and Ras signaling to induce EMT and tumor metastasis in human lung cancer cells. JMJD3 enzymatic activity may therefore be a better therapeutic target for human lung cancer.

## Results

### Induction of JMJD3 expression is correlated with poor prognosis in Ras-activated lung cancer cells

**Fig. 1:**
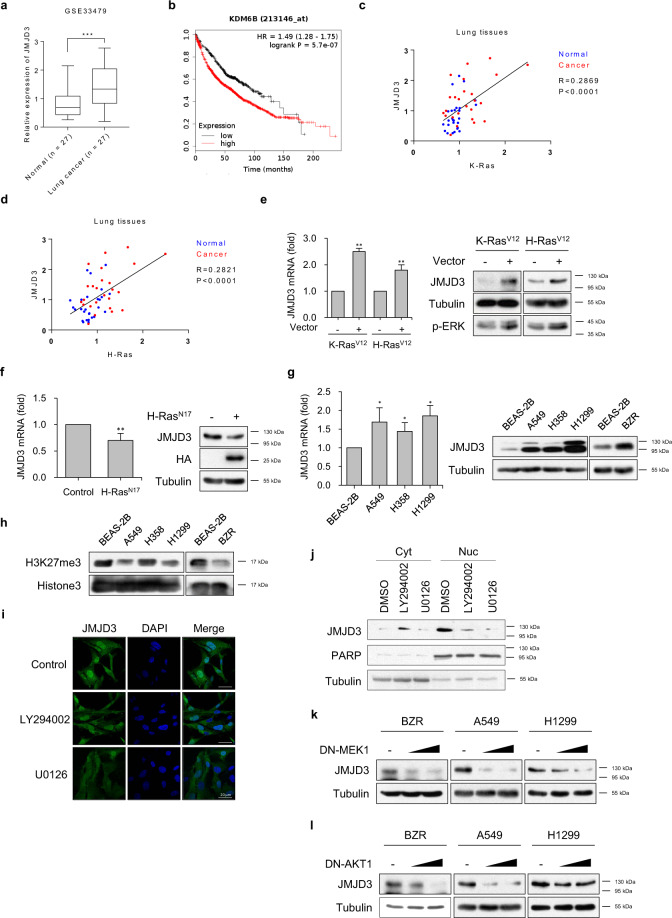
JMJD3 is overexpressed in Ras-activated lung cancer cells. **a** Relative expression of JMJD3 in lung tissues from lung cancer patients and normal (normal *n* = 27, lung cancer *n* = 27). ***P < 0.001 versus normal. **b** Overall survival rate curve of lung cancer patients according to JMJD3 expression (low *n* = 484, high *n* = 1442). **c** Correlative expression of JMJD3 compared to K-ras in lung tissues. **d** Correlative expression of JMJD3 compared to H-ras in lung tissues. **e** Real-time RT-PCR (left panel) and western blot analysis (right panel) of JMJD3 expression levels in BEAS-2B cells transfected with K-Ras^V12^ or H-Ras^V12^ expression vector. Columns, mean of three independent experiments performed in triplicate; *bar*, S.D.; **P < 0.01 versus control. **f** Real-time RT-PCR (left panel) and western blot analysis (right panel) of JMJD3 expression levels in BZR cells transfected with control or H-Ras^N17^ expression vector. Columns, mean of three independent experiments performed in triplicate; *bar*, S.D.; **P < 0.01 versus control. **g** Real-time RT-PCR (left panel) and western blot analysis (right panel) of JMJD3 expression levels in normal (BEAS-2B cells) and Ras-activated lung cancer cells (A549, H358, H1299, and BZR cells). Columns, mean of three independent experiments performed in triplicate; *bar*, S.D.; *P < 0.01 versus BEAS-2B. **h** Western blot analysis of H3K27me3 in BEAS-2B, A549, H358, H1299, and BZR cells. **i** Immunofluorescence analysis of expression and localization of JMJD3 (green) in A549 cells treated with DMSO, LY294002 (10 μM), or U0126 (10 μM). DAPI-staining nuclei (blue) were also shown. Scale bar, 20 μm. **j** Expression of JMJD3 in cytosolic or nuclear fractions of A549 cells treated with DMSO, LY294002 (10 μM), or U0126 (10 μM). PARP (nucleus) and tubulin (cytoplasm) were also shown. **k** Expression level of JMJD3 in BZR, A549, and H1299 cells transfected with the increasing amount of DN-MEK1 expression vector. **l** Expression level of JMJD3 in BZR, A549, and H1299 cells transfected with the increasing amount of DN-AKT1 expression vector.

In order to determine the role of JMJD3 in human lung cancer, we first explored human lung cancer patients within the GEO database and also examined several human lung cancer cell lines to determine whether abnormal JMJD3 expression existed. In the GEO database, human lung cancer patients had significant induction of JMJD3 mRNA levels compared to normal subjects (Fig. 1a). To evaluate whether differential expression of JMJD3 plays a role in human lung cancer progression, we explored the association between JMJD3 mRNA levels and overall survival in a cohort of human lung cancer patients from Kaplan–Meier plotter (KM Plotter). Patient survival was negatively associated with JMJD3 expression in lung cancer (Fig. 1b). We next found that the induction of JMJD3 was most highly correlated with K-Ras or H-Ras expression, particularly in lung tissue (Fig. 1c and d). In order to test whether JMJD3 is regulated by Ras activity, BEAS-2B cells (normal bronchial epithelial cells) were transfected with constitutively active (CA)-K-Ras (K-Ras^V12^) or CA-H-Ras (H-Ras^V12^) expression vectors, resulting in markedly elevated induction of JMJD3 mRNA and protein expression, as assessed by real-time RT-PCR and western blot (Fig. 1e, left and right panels). However, expression of dominant-negative (DN)-H-Ras (H-Ras^N17^) into BZR (H-Ras-transformed bronchial epithelial cells) suppressed JMJD3 expression (Fig. 1f, left and right panels). JMJD3 expression was consistently increased in Ras-activated lung cancer cells such as A549, H358, H1299, and BZR as compared with BEAS-2B cells (Fig. 1g).

JMJD3 is a histone demethylase with a preference for H3K27me3 which is responsible for removing methyl groups from H3K27. We measured the function of JMJD3 on histone demethylation in Ras-activated lung cancer cells. Histone extract analysis showed that H3K27me3 levels decreased when compared with BEAS-2B cells in RAS-activated lung cancer cells (Fig. 1h). Therefore, we were interested in whether JMJD3 might facilitate an epigenetic impact on the progression and metastasis of lung cancer based on oncogenic Ras signaling. To assess which signal pathway involves JMJD3 expression induced by Ras, we determined the effect of an ERK or PI3K inhibitor on JMJD3 expression in A549 cells. Immunofluorescence showed that ERK and PI3K inhibitors significantly abolished JMJD3 expression (Fig. 1i). We also confirmed the expression change in nucleus of JMJD3 through the cell nucleus fractionation assay. The expression of JMJD3 in nucleus decreased by ERK and PI3K inhibitors (Fig. 1j). Furthermore, dose-dependent expression of DN-MEK1 or DN-AKT1 into BZR or A549 or H1299 cells inhibited JMJD3 expression completely (Fig. 1k and l). We checked the cell migration and invasion activity in DN-MEK1 or DN-AKT1 transfected A549 cells (Supplementary Fig. 1a, b). MEK1 or AKT1-DN from transfected cells decreased cell migration and invasion compared to control cells. To find the transcription factor that could change the expression of JMJD3 in Ras downstream signaling, each of the transcription factors was knock-downed in A549 cells and found that Fra and c-Jun regulated the expression of JMJD3 as a transcription factor downstream of Ras (Supplementary Fig. 1c, d). These results indicated that the elevated expression of JMJD3 in Ras-activated human lung cancer cells plays an oncogenic role.

### JMJD3 enhances TGF-β-induced migration and EMT

**Fig. 2:**
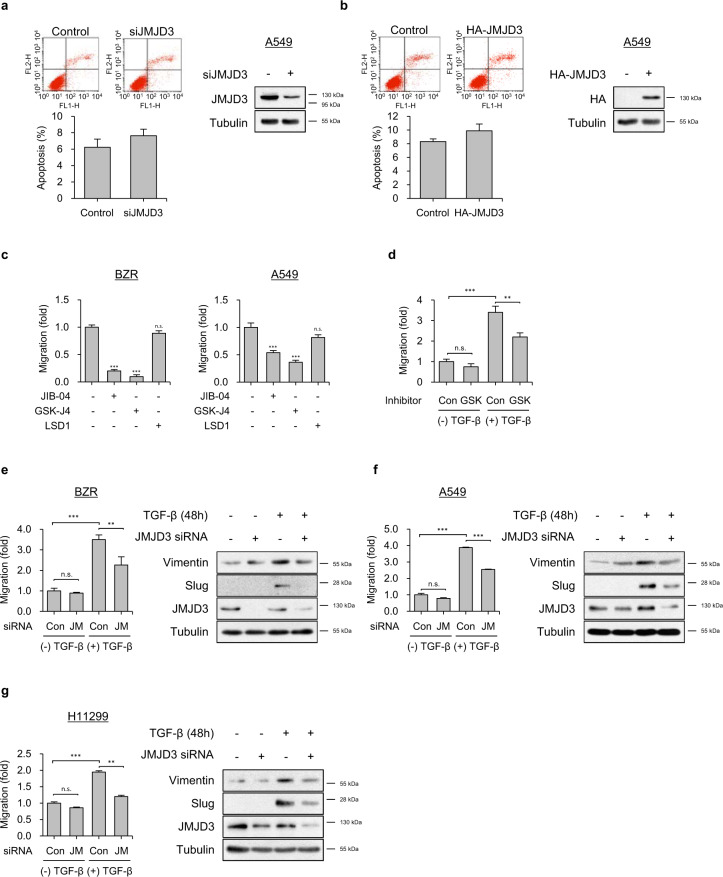
JMJD3 promotes TGF-β1-mediated migration and EMT in Ras-activated lung cancer cells. **a**, **b** Flow cytometry analysis for apoptosis in A549 cells transfected with **a** JMJD3 siRNA or **b** HA-JMJD3 expression vector. The cells were stained with Annexin V and PI, followed by analysis using flow cytometry. The percentage of apoptotic cells is shown in the bar graph. Whole-cell lysates were blotted with the indicated antibodies. **c** Transwell migration assay on BZR (left panel) or A549 (right panel) cells in response to JIB-04 (10 μM), GSK-J4 (10 μM), or LSD1 (10 μM). Columns, mean of two independent experiments performed in triplicate; *bar*, S.D.; ***P < 0.001 versus control, n.s.: nonsignificant. **d** Transwell migration assay on A549 cells in response to GSK-J4 (10 μM) in the presence or absence of TGF-β1 (5 ng/ml). Columns, mean of two independent experiments performed in triplicate; *bar*, S.D.; **P < 0.01, ***P < 0.001, n.s.: nonsignificant. **e**–**g** Transwell migration assay on **e** BZR, **f** A549, or **g** H1299 cells transfected with JMJD3 siRNA in the presence or absence of TGF-β1 (5 ng/ml) (left panel). Expression level of vimentin and slug, as assessed by western blot analysis (right panel). Columns, mean of two independent experiments performed in triplicate; *bar*, S.D.; **P < 0.01, ***P < 0.001 versus control, n.s.: nonsignificant.

JMJD3 is known to be a TGF-β1-induced EMT regulator through the induction of Slug as an EMT marker^33^. We were therefore interested in whether JMJD3 could have an epigenetic effect on the crosstalk between TGF-β1 and oncogenic Ras signaling that contributes to metastasis. In order to investigate the role of JMJD3 in Ras-activated tumorigenesis of lung cancer cells, we examined the effects of JMJD3 on cancer cell survival and migration. BZR cells infected with JMJD3 shRNA or A549 cells transfected with JMJD3 siRNA had no effect on cell proliferation (Supplementary Fig. 2a). Knockdown or overexpression of JMJD3 in A549 cells also did not have a significant effect on apoptosis (Fig. 2a and b). Similarly, overexpression of JMJD3 in BZR cells had no effect on apoptosis (Supplementary Fig. 2b). However, treatment with GSK-J4 (a specific JMJD3 inhibitor) abolished the cell migration of BZR and A549 cells (Fig. 2c).

To further determine whether JMJD3 was required for TGF-β1-induced migration in Ras-activated lung cancer cells, we utilized GSK-J4 and siRNA to knockdown JMJD3 expression in BZR, A549, and H1299 cells. GSK-J4 suppressed TGF-β1-induced cell migration (Fig. 2d). In addition, suppression of JMJD3 significantly inhibited TGF-β1-induced cell migration and EMT marker protein expressions such as vimentin and Slug in BZR, A549, and H1299 cells (Fig. 2e–g). H-Ras^N17^-overexpressing BZR or H-Ras^N17^-overexpressing A549 cells suppressed TGF-β1-induced E-cadherin loss and increases in mesenchymal marker proteins such as vimentin and Slug (Supplementary Fig. 2c). These data indicated that JMJD3 might play a key role in linking Ras activity with EMT transition induced by TGF-β in lung cancer cells.

### JMJD3 enhances TGF-β-induced Smad2/3 activation

**Fig. 3:**
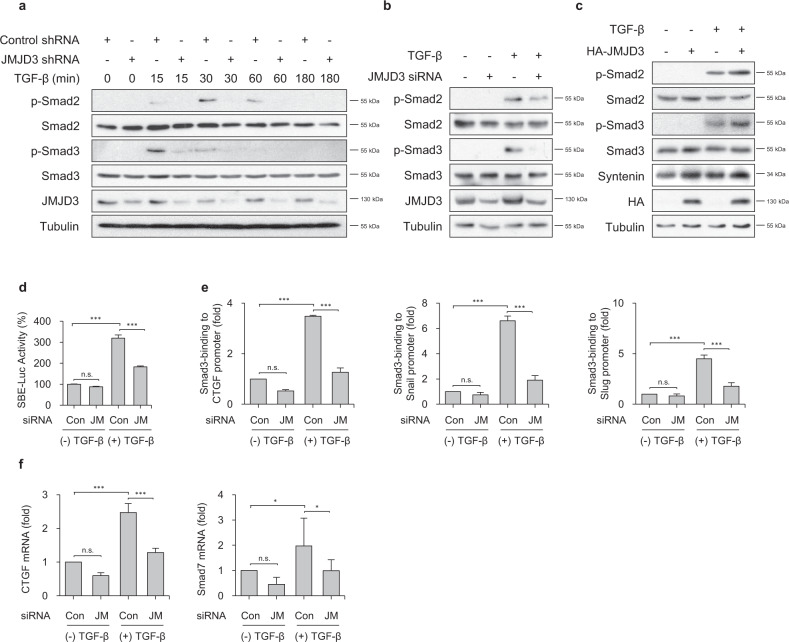
JMJD3 enhances TGF-β1-induced Smad2 and Smad3 activation via induction of syntenin. **a** Phosphorylation of endogenously expressed smad2 and smad3 in BZR cells transfected with control shRNA or JMJD3 shRNA in response to TGF-β1 (5 ng/ml) for the indicated periods of time. **b** Expression level of p-smad2 and p-smad3 in A549 cells transfected with JMJD3 siRNA in response to TGF-β1 (5 ng/ml) for 1 h. **c** Expression level of p-smad2 and p-smad3 in BEAS-2B cells transfected with HA-tagged JMJD3 expression vector in response to TGF-β1 (5 ng/ml) for 1 h. **d** Luciferase activity of SBE-Luc reporter plasmid-expressed BZR cells transfected with control siRNA or JMJD3 siRNA in response to TGF-β1 (5 ng/ml) for 24 h. Columns, mean of three independent experiments performed in triplicate; *bar*, S.D.; ***P < 0.001 versus control, n.s.: nonsignificant. **e** Chromatin immunoprecipitation (ChIP) assay to analyze smad3-binding to target promoters such as CTGF (left panel), snail (middle panel), and slug (right panel) in BZR cells transfected with control siRNA or JMJD3 siRNA in response to TGF-β1 (5 ng/ml) for 1 h. Columns, mean of two independent experiments performed in triplicate; *bar*, S.D.; ***P < 0.001 versus control, n.s.: nonsignificant. **f** Real-time RT-PCR of CTGF (left panel) and smad7 (right panel) expression levels in A549 cells transfected with control siRNA or JMJD3 siRNA in response to TGF-β1 (5 ng/ml) for 12 h. Columns, mean of three independent experiments performed in triplicate; *bar*, S.D.; *P < 0.05, ***P < 0.001 versus control, n.s.: nonsignificant.

We examined whether JMJD3 regulates TGF-β-induced Smad2 and Smad3 activation, as these are key transcriptional activators of canonical TGF-β1 signaling pathway. JMJD3 knockdown by shRNA decreased TGF-β1-induced phosphorylation of Smad2 and Smad3 in BZR cells (Fig. 3a). JMJD3 knockdown in A549 cells also impaired TGF-β1-induced Smad2 and Smad3 phosphorylation (Fig. 3b). However, overexpression of JMJD3 in BEAS-2B cells promoted TGF-β1-induced Smad2 and Smad3 phosphorylation (Fig. 3c). In parallel with suppression of TGF-β1-induced Smad Binding Element (SBE)-Luc gene, the binding of Smad3 to target gene promoters including CTGF, Snail, and Slug was dramatically reduced in JMJD3-depleted BZR cells (Fig. 3d and e). In addition, Smad3 target genes such as CTGF and Smad7 were abolished in JMJD3-depleted BZR cells in response to TGF-β1 stimulation (Fig. 3f).

We investigated whether the Ras-JMJD3 axis affects TGF-β1-induced Smad2/3 activation. In BZR cells, TGF-β-induced phosphorylation of Smad2 and Smad3, as compared to BEAS-2B cells is significantly increased (Supplementary Fig. 3a). Overexpression of Ras^N17^ in BZR cells reduced TGF-β1-induced phosphorylation of Smad2 and Smad3 (Supplementary Fig. 3b). A reporter gene assay using SBE-Luc also revealed that in BZR cells, the gene expression level of TGF-β1-induced SBE-Luc, as compared with BEAS-2B cells is increased (Supplementary Fig. 3c). Binding of Smad3 to CTGF promoter was significantly increased in BZR cells in response to TGF-β1 stimulation (Supplementary Fig. 3d), as assessed by ChIP assay. We also confirmed that treatment of BZR cells with TGF-β1 enhanced upregulation of Slug and downregulation of E-cadherin, as compared with BEAS-2B cells (Supplementary Fig. 3e).

### JMJD3 epigenetically induces syntenin expression

**Fig. 4:**
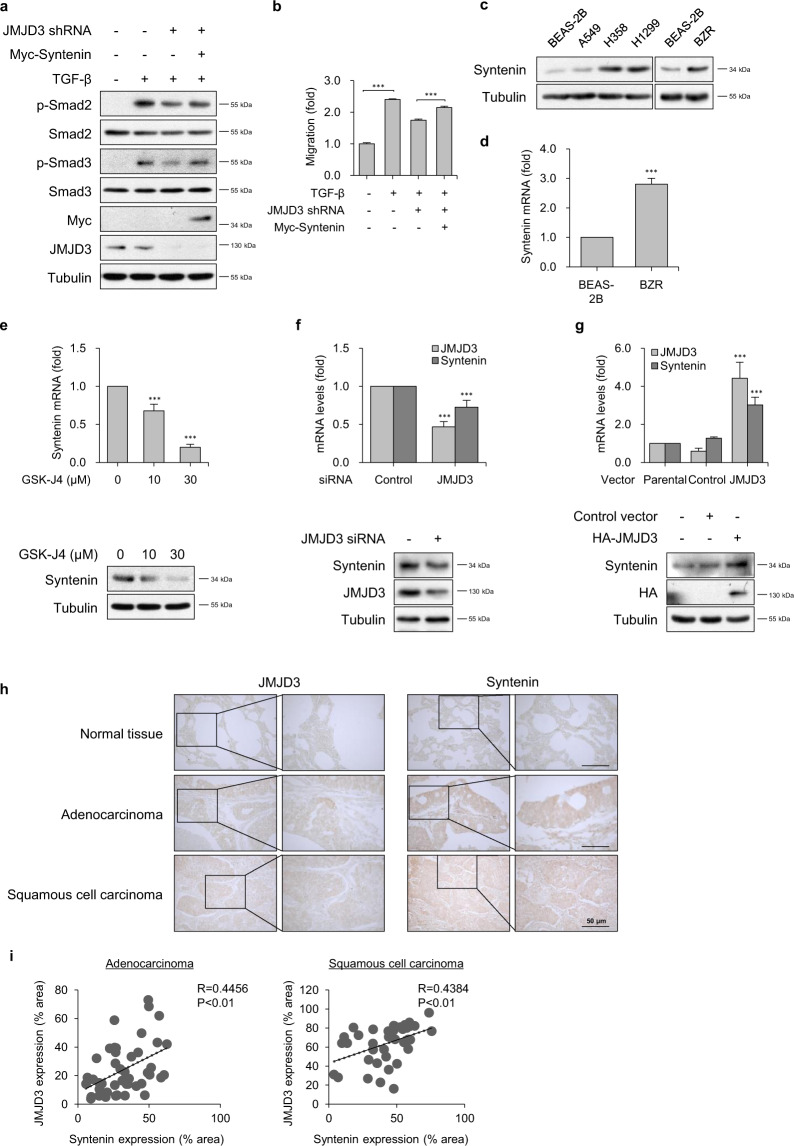
JMJD3 epigenetically induces expression of syntenin in Ras-activated lung cancer cells. **a** Expression level of p-smad2, p-smad3 in BZR cells transfected with the indicated plasmids in response to TGF-β1 (5 ng/ml) for 1 h. **b** Transwell migration assay on BZR cells transfected with the indicated plasmids in response to TGF-β1 (5 ng/ml). Columns, mean of two independent experiments performed in triplicate; *bar*, S.D.; ***P < 0.001 versus control. **c** Syntenin protein expression in BEAS-2B, A549, H358, H1299, and BZR cells. **d** Real-time RT-PCR analysis of syntenin mRNA levels in BEAS-2B and BZR cells. Data are presented as the mean±s.d. of three independent experiments; *bar*, S.D.; ***P < 0.001 versus BEAS-2B. **e** Real-time RT-PCR analysis (upper panel) and western blot analysis (lower panel) of syntenin expression in A549 cells in response to GSK-J4. Columns, mean of three independent experiments performed in triplicate; *bar*, S.D.; ***P < 0.001 versus control. **f** Real-time RT-PCR analysis (upper panel) and western blot analysis (lower panel) of JMJD3 and syntenin expression levels in BZR cells in response to knockdown of JMJD3. Columns, mean of three independent experiments performed in triplicate; *bar*, S.D.; ***P < 0.001 versus control. **g** Real-time RT-PCR analysis (upper panel) and western blot analysis (lower panel) of JMJD3 and syntenin expression levels in BEAS-2B cells transfected with control or HA-tagged JMJD3. Columns, mean of three independent experiments performed in triplicate; *bar*, S.D.; ***P < 0.01 versus control. **h** A representative image of immunohistochemical staining with JMJD3 and syntenin in human lung cancer tissue microarray. Scale bar, 50 μm. **i** The positive correlation between JMJD3 and syntenin expression in human lung cancer tissues. Pearson’s coefficient tests were performed to assess significance.

We recently showed that syntenin overexpression facilitated TGF-β1-mediated Smad2/3 activation and EMT by inhibiting caveolin-mediated TβRI internalization and thereby increasing plasma-membrane localization of TβRI^35^. However, the mechanism(s) by which syntenin regulates TβRI-mediated Smad2/3 phosphorylation and activation in lipid rafts at the plasma membrane has not been investigated. We therefore explored whether JMJD3 affects syntenin-regulated Smad2 and Smad3 phosphorylation with TGF-β1 stimulation. Overexpression of syntenin in stable JMJD3-knockdown BZR cells significantly recovered down-regulated TGF-β1-induced Smad2 and Smad3 phosphorylation and cell migration (Fig. 4a and b). These results suggest that Ras-activated JMJD3 enhances TGF-β1-induced Smad2 and Smad3 activation through induction of syntenin expression.

We found that, as for JMJD3, the expression level of syntenin was significantly increased in BZR, A549, H358, and H1299 cells, as compared with BEAS-2B cells (Fig. 4c and d). K-Ras^V12^ or H-Ras^V12^-overexpressing BEAS-2B cells increased expression of syntenin at both mRNA and protein levels (Supplementary Fig. 4a). However, H-Ras^N17^-overexpressing BZR cells reduced expression of syntenin (Supplementary Fig. 4b). DN-MEK or DN-AKT1 in BZR, A549, and H1299 cells significantly inhibited syntenin expression in a dose-dependent manner (Supplementary Fig. 4c and d). To investigate whether JMJD3 could specifically control expression of syntenin, we examined the effect of GSK-J4 on syntenin expression in A549 cells. Dose-dependent treatment with GSK-J4 significantly reduced syntenin mRNA and protein expression (Fig. 4e). Similarly, GSK-J4 treatment in BZR cells suppressed syntenin expression (Supplementary Fig. 4e), In addition, JMJD3 knockdown impaired syntenin expression (Fig. 4f). However, overexpression of JMJD3 significantly increased syntenin mRNA and protein expression compared with control, suggesting JMJD3 may regulate syntenin expression (Fig. 4g).

To clarify the expression profiles of JMJD3 and syntenin and to explore the clinical implications of JMJD3 expression in human lung cancer, we performed immunohistochemical staining for JMJD3 and syntenin with human lung tissue microarrays. Our results showed increased nuclear expression of JMJD3 and cytoplasmic expression of syntenin in human lung cancer tissue including adenocarcinoma or squamous cell carcinoma compared to normal tissues (Fig. 4h). JMJD3 and syntenin expression levels exhibited a significant positive correlation in adenocarcinoma or squamous cell carcinoma tissues (Fig. 4i).

### JMJD3 binds to the syntenin promoter and demethylates H3K27me3

**Fig. 5:**
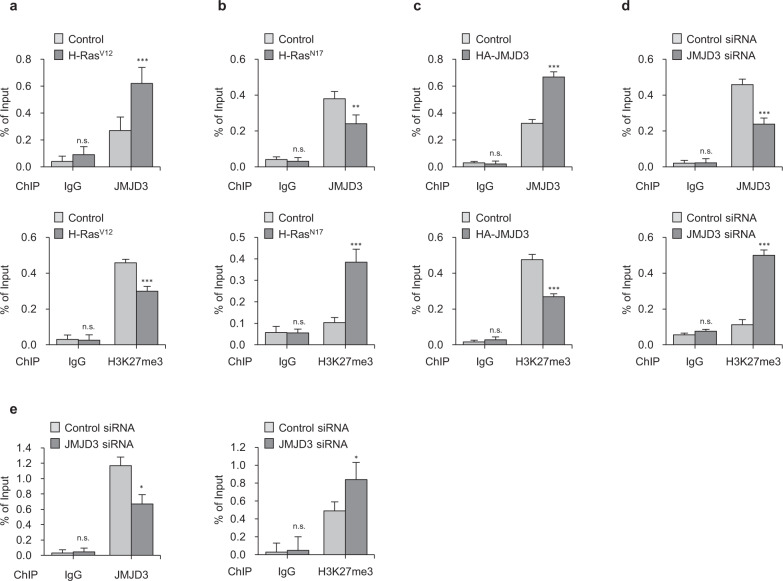
JMJD3 binds to syntenin promoter and demethylates H3K27me3. **a** ChIP assay of JMJD3 (upper panel) and H3K27me3 (lower panel) recruitment at syntenin promoter using IgG, JMJD3, or H3K27me3 antibody in H-Ras^V12^-expressed BEAS-2B cells. Columns, mean of two independent experiments performed in triplicate; *bar*, S.D.; ***P < 0.001 versus control in each group, n.s.: nonsignificant. **b** ChIP assay of JMJD3 (upper panel) and H3K27me3 (lower panel) recruitment at syntenin promoter using IgG, JMJD3, or H3K27me3 antibody in H-Ras^N17^-expressed BZR cells. Columns, mean of two independent experiments performed in triplicate; *bar*, S.D.; **P < 0.01, ***P < 0.001 versus control in each group, n.s.: nonsignificant. **c** ChIP assay of JMJD3 (upper panel) and H3K27me3 (lower panel) recruitment at syntenin promoter using IgG, JMJD3, or H3K27me3 antibody in HA-JMJD3-expressed BEAS-2B cells. Columns, mean of two independent experiments performed in triplicate; *bar*, S.D.; ***P < 0.001 versus control in each group, n.s.: nonsignificant. **d** ChIP assay of JMJD3 (upper panel) and H3K27me3 (lower panel) recruitment at syntenin promoter using IgG, JMJD3, or H3K27me3 antibody in BZR cells in response to JMJD3 knockdown. Columns, mean of two independent experiments performed in triplicate; *bar*, S.D.; ***P < 0.001 versus control in each group, n.s.: nonsignificant. **e** ChIP assay of JMJD3 (upper panel) and H3K27me3 (low**e**r panel) recruitment at syntenin promoter using IgG, JMJD3, or H3K27me3 antibody in A549 cells in response to JMJD3 knockdown. Columns, mean of two independent experiments performed in triplicate; *bar*, S.D.; *P < 0.05 versus control in each group, n.s.: nonsignificant. *P < 0.05 versus control in each group, n.s.: nonsignificant.

To test the hypothesis that JMJD3 regulates syntenin expression by erasing H3K27me3, we first investigated whether the translocated JMJD3 interacts with the syntenin promoter region. Four primer sets for the promoter region near the transcription start site were synthesized for ChIP assays. The ChIP analyses demonstrated that JMJD3 binds to the human syntenin transcriptional start region, but not to the promoter region upstream of the transcription start site. Binding to the primer 4 region was concentration-dependent, as determined by ChIP assays using anti-JMJD3 antibody as well as H3K28me3 antibody. JMJD3 binds to the primer 4 region of syntenin in BZR cells but not in BEAS-2B cells (Supplementary Fig. 5a, Fig. 5b, c). ChIP-qPCR analysis showed that recruitment of JMJD3 into the syntenin promoter region in BEAS-2B cells expression of H-Ras^V12^ was increased, leading to a dramatic reduction in H3K27me3 as compared to control cells (Fig. 5a). In contrast, overexpression of H-Ras^N17^ in BZR cells resulted in decreased JMJD3 binding and increased H3K27me3 enrichment at the syntenin promoter region (Fig. 5b).

To validate that JMJD3 could specifically bind to the syntenin promoter and regulate H3K27me3 patterns, HA-tagged JMJD3 was expressed in BEAS-2B cells, then ChIP-qPCR revealed that the recruitment of JMJD3 to the syntenin promoter was significantly increased, in parallel with reductions in H3K27me3 enrichment (Fig. 5c). Knockdown of JMJD3 with BZR cells, however, markedly decreased recruitment of JMJD3 to syntenin promoter in parallel with dramatically increased H3K27me3 enrichment. Similarly, JMJD3 knockdown in A549 cells decreased JMJD3 binding and increased H3K27me3 enrichment at the syntenin promoter (Fig. 5d, e). Taken together, these findings suggest that JMJD3 binds directly to syntenin promoter and regulates syntenin expression by impairing H3K27me3.

### JMJD3 enhances TGF-β-induced EMT by increasing syntenin-mediated TβRI/Smad2/3 complex formation

**Fig. 6:**
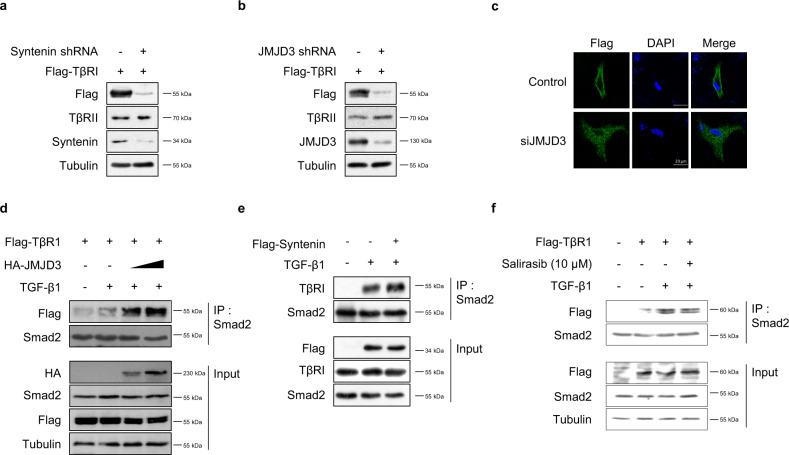
JMJD3 promotes TGF-β-induced EMT by increasing syntenin-mediated formation of TβRI/Smad2/3 complex. **a** Expression of TβRI and TβRII protein expression in BZR cells transfected with Flag-TβRI and/or syntenin shRNA. **b** Expression of TβRI and TβRII protein expression in BZR cells transfected with Flag-TβRI and/or JMJD3 shRNA. **c** Immunofluorescence analysis of expression and localization of Flag-TβRI (green) in A549 cells in response to knockdown of JMJD3. DAPI-staining nuclei (blue) were also shown. Scale bar, 20 μm. **d** Immunoprecipitates (IP) analysis of protein interactions between TβRI and smad2 in A549 cells transfected with the increasing amounts of HA-tagged JMJD3 expression vector (1 and 3 µg) in response to TGF-β1 (5 ng/ml) for 1 h. **e** IP analysis of protein interactions between TβRI and smad2 in BZR cells transfected with Flag-syntenin expression vector in response to TGF-β1 (5 ng/ml) for 1 h. **f** IP analysis of protein interactions between TβRI and smad2 in Flag-TβR1-expressed A549 cells treated with Salirasib (10 μM) for 24 h in response to either and TGF-β1 (5 ng/ml) for 1 h.

To elucidate the mechanisms by which Ras-JMJD3-syntenin axis is involved in TGF-β1-induced Smad signaling and EMT, we confirmed the effect of JMJD3 or syntenin depletion on expression levels of Flag-tagged TβRI. Stable knockdown of JMJD3 or syntenin by shRNA in BZR cells decreased the expression level of Flag-tagged TβRI, but not that of TβRII (Fig. 6a, b). Knockdown of JMJD3 in A549 cells also decreased expression of TβRI on the plasma membrane, as assessed by immunofluorescence (Fig. 6c). Using immunoprecipitation, we further examined whether JMJD3 modulates the formation of the TGF-β-induced TβRI/Smad2/3 complex, which is an essential step for TβRI-mediated Smad2/3 phosphorylation. Overexpression of JMJD3 significantly promoted TGF-β1-induced TβRI/Smad2/3 complex formation in a dose-dependent manner (Fig. 6d). Similarly, overexpression of syntenin increased the TGF-β1-induced TβRI/Smad2/3 complex formation in BZR cells (Fig. 6e). In addition, we examined the effect of Salirasib as a Ras inhibitor on TGF-β1-induced formation of TβRI/Smad2/3 complex. Treatment of Salirasib significantly suppressed the TGF-β1-induced recruitment of Smad2/3 to TβRI (Fig. 6f). To investigate Ras-TGF-β1-EMT correlation, we performed the cell migration assay in BZR and Beas2B cells (Supplementary Fig. 6a and Fig. 6b). TGF-β1 signaling inhibitors reduced cell migration activity induced by TGF-β1 or active Ras. These results suggest that Ras-activated JMJD3 plays a critical role in TGF-β1-induced EMT by promoting the formation of syntenin-mediated TβRI/Smad2/3 complex.

### Inhibition of JMJD3 activity reduced tumor growth in vivo

To ensure that JMJD3 regulates tumor growth in vivo, we performed tumor xenograft in mice. We induced the formation of tumor by injecting H1299 cells into mice, and started treatment with JMJD3 inhibitor GSK-J4 or DMSO as a control in mice after 4 weeks (Supplementary Fig. 7a). The tumor volume decreased meaningfully in mice treated with the GSK-J4 compared to control mice at 36 and 38 days (Supplementary Fig. 7b). The tumor size at 42 days was smaller in GSK-J4 treated mice than control mice (Supplementary Fig. 7c). In addition, we checked the expression of p-smad2 using immunohistochemistry assay in tumor tissue. Entire p-smad2 expression reduced in tumor from GSK-J4-treated mice relative to controls. In particular, nuclear expression of p-smad2 was significantly diminished in GSK-J4-treated group (Supplementary Fig. 7d). These results show that reduced JMJD3 activity inhibited tumor growth in vivo.

## Discussion

In response to TGF-β1, H-Ras-transformed cells exhibit enhanced metastatic behavior. TGF-β1 induces EMT of mammary-derived tumors and metastasis of epithelial tumor cells, in combination with activated H-Ras^13^. In addition, H-Ras modulates EMT and metastasis by TGF-β1-regulated Smad2 nuclear accumulation in spindle tumor cells^36^. TGF-β1 can activate H-Ras signaling in epithelial cells, and contributes to tumor progression by enhancing cell migration and invasion through downstream H-Ras signaling, for example, p38 MAPK and ERK1/2 in human breast epithelial cells^14^. Recent studies have shown that cooperation of TGF-β1 and H-Ras promotes EMT and metastasis in diverse tumor cells; however, the mechanism(s) of cooperation between TGF-β1 and H-Ras signaling is still not fully understood. Here, we demonstrated that JMJD3 histone H3K27 demethylase plays an important role in the collaboration between TGF-β signaling and oncogenic RAS for activation of Smad2/3 and EMT in human lung cancer cells. Mechanistically, we provide evidence showing that JMJD3 induced by Ras activation is not only recruited directly to the syntenin promoter but also that there is demethylation of H3K27me3 and increased syntenin expression. In consequence, JMJD3 enhanced formation of the TβRI/ Smad2/3 complex mediated by syntenin, which is required for Smad2/3 activation. This process facilitated the promotion of TGF-β-induced EMT and metastasis. In addition, we provide important new insights into how crosstalk between TGF-β and H-Ras signaling induces EMT and tumor metastasis in human lung cancer cells through activation of JMJD3, and highlight that JMJD3 might be a new therapeutic target against Ras-activated lung cancer cells.

We identified JMJD3 as a strong enhancer of TGF-β signaling in Ras-activated lung cancer cells and showed that it induces syntenin to promote the activation of Smad2/3, thereby promoting tumorigenic TGF-β function in EMT and metastasis. On the other hand, it has been reported that JMJD3 functions as a tumor suppressor and inhibits tumor progression including cell proliferation and metastasis^37–39^. Overexpression of JMJD3 induces mitochondria-dependent apoptosis and inhibits cell growth in non-small cell lung cancer. In contrast, knockdown of JMJD3 elicited cell growth and suppressed apoptosis^40^. However, our study revealed that both overexpression and knockdown of JMJD3 have no effect on cell viability and apoptosis in non-small lung cancer cells including A549 cells. Moreover, inhibition of JMJD3 by GSK-J4, a specific JMJD3 inhibitor, abolished cell migration in the absence and presence of TGF-β in A549 cells. Thereby, we suggest that JMJD3 may have higher oncogenic functions than tumor suppressor activity in Ras-activated lung cancer cells.

JMJD3 is significantly increased in lung cancer tissues but is rarely expressed in normal tissues, as confirmed by human lung cancer tissue microarray. We found that JMJD3 is highly expressed in Ras-activated lung cancer cells. Previous studies have shown that the RAS/RAF/MEK/ERK pathway controls the expression of JMJD3. Activation of ERK phosphorylates transcription factors including c-Jun and c-Fos in the nucleus, suggesting that ERK induces JMJD3 through the transcription factor AP-1^29^. While we found that oncogenic Ras induced the expression levels of JMJD3 via Ras-ERK or Ras-AKT pathways, the detailed mechanism(s) by which Ras activity regulates JMJD3 expression needs to be studied further.

Interestingly, syntenin expression in several cancer cells has been shown to be increased, although the molecular mechanisms underlying Ras-regulated syntenin expression have not been investigated. Our findings suggest that Ras signaling increases JMJD3 expression, and then JMJD3 induces syntenin expression. In consequence, syntenin increases TGF-β1-induced Smad2/3 activation and EMT transition in cancer metastasis. We provide the first evidence that JMJD3 epigenetically regulates the expression levels of syntenin via H3K27me3 demethylation, leading to TGF-β1-induced Smad2/3 activation and EMT in lung cancer cells. JMJD3 functions as a molecular linker to cooperate TGF-β1 and oncogenic Ras pathways for inducing EMT through regulation of syntenin expression. A previous study showed that JMJD3 promotes the cell migration, invasion, and metastasis of ovarian cancer cells by modulating TGF-β1 expression^41^. Therefore, we need to perform further studies to confirm the promotive effect of JMJD3 on TGF-β1 signaling in Ras-activated lung cancer cells. This study proposes a model of molecular crosstalk between TGF-β and Ras signaling in human lung cancer. Importantly, JMJD3 histone H3K27 demethylase may act as a critical collaborator and promote tumor progression via regulating the expression of syntenin. JMJD3 might serve as an effective target in combined therapeutics against TGF-β and oncogenic Ras signaling.

## Materials and methods

### Cell culture

Immortalized human bronchial epithelial BEAS-2B cells, H-Ras transformed human bronchial epithelial BZR cells and human non-small cell lung carcinoma A549, H358, and H1299 cells were purchased from American Type Culture Collection (ATCC). BZR cells were cultured in DMEM supplemented with 10% heat-inactivated fetal bovine serum and 1% penicillin-streptomycin solution (Invitrogen, Carlsbad, CA, USA). BEAS-2B, A549, H358, and H1299 cells were cultured in RPMI-1640 supplemented with 10% heat-inactivated fetal bovine serum and 1% penicillin-streptomycin solution. All cells were maintained in a humidified 5% CO_2_ atmosphere at 37 °C.

### Immunoblotting, immunoprecipitation, immunofluorescence, immunohistochemistry, reagents, and antibodies

The experimental procedures were performed as previously described^35^. The antibodies used are listed in Supplementary Table S1. The reagents used are listed in Supplementary Table S2.

### Plasmids, RNA interference, and small hairpin RNA

The expression vectors for Myc- and Flag-tagged syntenin were described previously^42,43^. The H-Ras^V12^, K-Ras^V12^, H-Ras^N17^, and DN-MEK and DN-AKT constructs were kindly provided by Dr. KY Lee (Cheonnam National University, Kwangju, Korea). TGF-β1-responsive luciferase reporter plasmid, SBE-Luc, and the expression vector for Flag-TβRI were kindly provided by Dr. BC Kim (Kangwon National University, Chuncheon, Korea). The expression vector for JMJD3 was purchased from KRIBB (Daejon, Korea). Small interfering RNA (siRNA), control siRNA, and lentiviral-based small hairpin RNA (shRNA) for syntenin and JMJD3 were purchased from Origene Technologies (Rockville, MD, USA). Small interfering RNA (siRNA) for c-Fos, Fra1, and c-Jun purchased from Bioneer (Daejeon, Korea). Lentiviral particles were prepared according to the manufacturer’s instructions (Origene Technologies, USA). Transfections were performed using Lipofectamine Plus reagent according to the manufacturer’s instructions (Invitrogen). After transfection for 48 h, the cells were used in experiments.

### Real-time quantitative polymerase chain reaction (qPCR)

Total RNAs were isolated using RNeasy mini kits according to the manufacturer’s instructions (Qiagen, Santa Clarita, CA, USA). Total RNA (1 μg) was used to synthesize cDNA using a Maxime RT PreMix Kit (Intron Biotechnology, Daejeon, Korea). Real-time qPCR was performed on a StepOne Real-time PCR System (Applied Biosystems, Foster City, CA, USA). The primers are listed in Supplementary Table S3.

### Chromatin immunoprecipitation (ChIP) assay

Chromatin immunoprecipitation (ChIP) assay procedures were performed as previously described^44^. The primers used for the ChIP assay are listed in Supplementary Table S4.

### Reporter gene assay

Cells were harvested and lysed with luciferase lysis buffer, and then assayed using the Dual-luciferase reporter assay system following the manufacturer’s instructions (Promega, Madison, WI, USA).

### Apoptosis assay

Cell apoptosis assays were performed using the Annexin V-FITC apoptosis detection kit according to the manufacturer’s instructions (BD Biosciences, CA, USA). Cells were collected, washed with cold PBS, and then stained with Annexin V-FITC and 2 µg/mL PI (propidium iodide) in the dark for 15 min at 37 °C. The samples were analyzed by flow cytometry using a FACS Calibur flow cytometer (Becton Dickinson, CA, USA).

### Proliferation assay

Proliferation assay was determined by MTT (3-(4,5-dimethylthiazol-2-yl)-2,5-diphenyltetrazolium bromide)-based colorimetric assay. MTT was purchased from Sigma-Aldrich. In brief, cells were seeded into 96-well plates with a density of 2 × 10^4^/well in triplicate. After incubation for 48 or 72 h, MTT solution (5 mg/mL) was added to each well and further incubated for 4 h at 37 °C. Cell proliferation was determined by measuring the absorbance at 570 nm.

### Cell migration and invasion assay

Cell migration assays were performed using the Boyden chamber method and polycarbonate membranes with an 8-μm pore size, as described previously^35^. Migrated cells on the lower surface of the filter were fixed with 4% formaldehyde. The cells were stained with H&E or DAPI and then counted in at least five randomly selected microscopic fields (×100) per filter.

### Nuclear and cytoplasmic fractionation assay

Cell fractionation assay was performed using a NE-PER nuclear and cytoplasmic extraction kit (Thermo Scientific) in accordance with the manufacturer’s protocol.

### Animal model in mice

Four-week female athymic nude mice were used (Orient Bio, Korea). Cell lines were trypsinized, washed, and counted using the Trypan blue solution (Sigma). For studies with H1299 Parental cells, injections were done with 10^7^ cells suspended in PBS were injected subcutaneously into the right flank of mice. Tumor growth was monitored by caliper measurements and tumor volume was calculated as (0.5 × length × width^2^). Treatment was started when tumors reached about 100–150 mm^3^, mice are randomized into control and treatment either a vehicle or GSK-J4. Mice were given vehicle or 100 mg/kg GSK-J4 (Tocris, dissolved in 1:1:8 = GSK-J4:chremopore:PBS) with blinding, given intraperitoneal, every day for 7 consecutive days, as documented previously (Hashizume et al.^45^). After 42 days, mice were sacrificed, and tumors were removed and embedded in OCT compound. Tissue samples were sectioned at a thickness of 5 μm. For antigen retrieval, the slides were pretreated by heating at 95 °C for 5 min in a retrieval buffer of Tris-EDTA buffer contained with Tween 20 (pH 8.0). The tumor tissues were performed immunohistochemistry analysis using the HRP-DAB Cell & Tissue Staining Kit (R&D Systems, Inc., McKinley Place NE Minneapolis, USA). Representative images of immunostaining results are shown. All animal studies were conducted in accordance with procedures approved by the Institutional Animal Care and Use Committee (IACUC) of Kangwon National University (IACUC approval No. KW-200320-2).

### Bioinformatics analyses with KM data and GEO data

A Kaplan–Meier plot was constructed by KM plotter website (https://kmplot.com/analysis/) to estimate the overall survival. The probe that traced the expression of JMJD3 was ‘213146_at’. The mRNA expression level data from the National Center for Biotechnology Information (National Institutes of Health, Bethesda, MD, USA) Gene Expression Omnibus database (GEO; http://www.ncbi.nlm.nih.gov/geo/) were downloaded. The GSE 33479 number database was used and the data platform was Agilent-014850 Whole Human Genome Microarray. Microarray expression data were obtained from the GEO33479 dataset in 27 lung cancer patients and 27 normal controls. To assess significant differences between sample groups, the unpaired Student’s *t* test per gene probe was used. Correlation coefficient *r* and *p* values are shown.

### Statistics

Data analysis was conducted with GraphPad Prism® version 7.01. The statistical differences were measured with unpaired two-tailed Student’s *t* test, when only two groups were compared. Otherwise, statistical significance was determined using two-way ANOVA followed by Bonferroni multiple comparison test. *P* value < 0.05 was considered statistically significant.

## Supplementary information

Epigenetic regulation of TGF-β-induced EMT by JMJD3/KDM6B histone H3K27 demethylase

Epigenetic regulation of TGF-β-induced EMT by JMJD3/KDM6B histone H3K27 demethylase
