# Supplementary material for: Epigenetic regulation of TGF-β-induced EMT by JMJD3/KDM6B histone H3K27 demethylase
Source: Oncogenesis. 2021 Feb 26;10(2):17. doi: 10.1038/s41389-021-00307-0 (PMC7910473; doi:10.1038/s41389-021-00307-0)
Supplement: Supplementary file 1 — Epigenetic regulation of TGF-β-induced EMT by JMJD3/KDM6B histone H3K27 demethylase [file 41389_2021_307_MOESM1_ESM.docx]

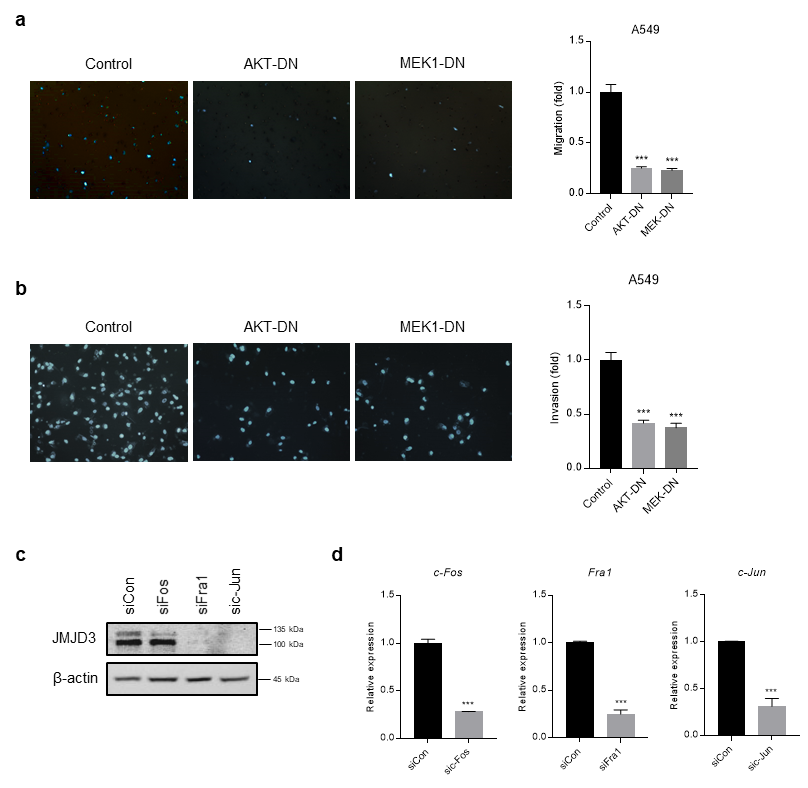


**Supplementary figure 1. Ras activity regulates JMJD3 expression and the ability of cell migration and invasion.** (**a**) Transwell migration assay and (**b**) invasion assay on A549 cells transfected with AKT-DN or MEK1-DN expression vector. Representative images with DAPI staining of transwell membrane (left panel). Columns, mean of three independent experiments performed in triplicate; *bar*, S.D.; ***P < 0.001 versus control. (**c**) Expression level of JMJD3 in A549 cells transfected with control, c-Fos, Fra1 or c-Jun siRNA. (**d**) Real-time RT-PCR analysis of c-Fos, Fra1 and c-Jun mRNA levels. Columns, mean of three independent experiments performed in triplicate; *bar*, S.D.; ***P < 0.001 versus siCon.


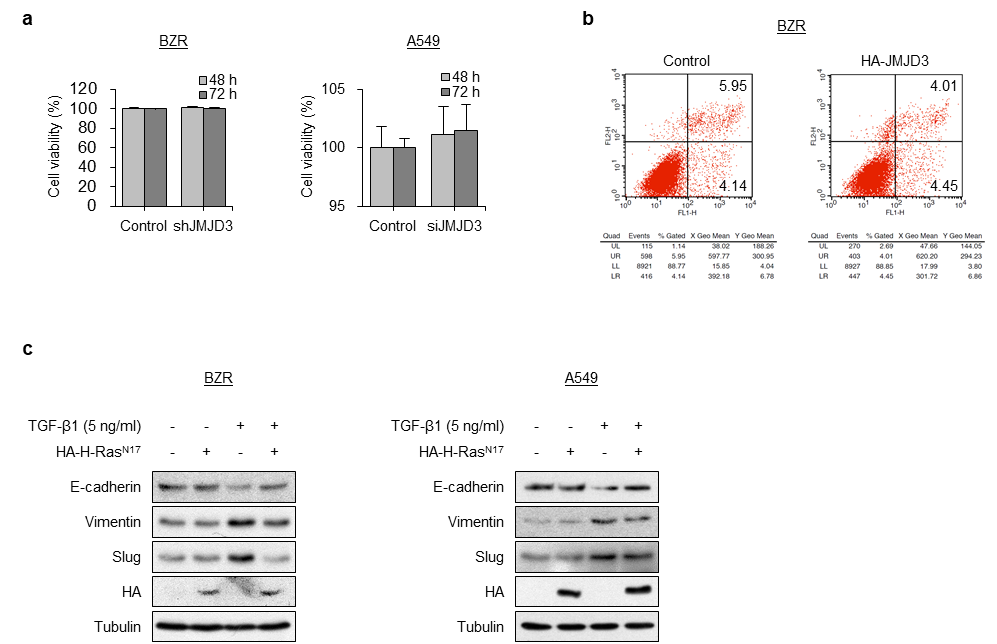


**Supplementary figure 2. Ras-JMJD3 axis promotes TGF-β1-mediated migration and EMT in Ras-activated lung cancer cells, but has no effect on cell proliferation.** (**a**) MTT assay in BZR or A549 cells transfected with control siRNA or JMJD3 siRNA. (**b**) Flow cytometry analysis for apoptosis in BZR cells transfected with control vector or HA-JMJD3 vector. (**c**) Expression level of E-cadherin, vimentin, slug in BZR cells (left panel) or A549 cells (right panel) transfected with HA-tagged H-RAS^N17^ in response to TGF-β1 (5 ng/ml) for 24 h.

**
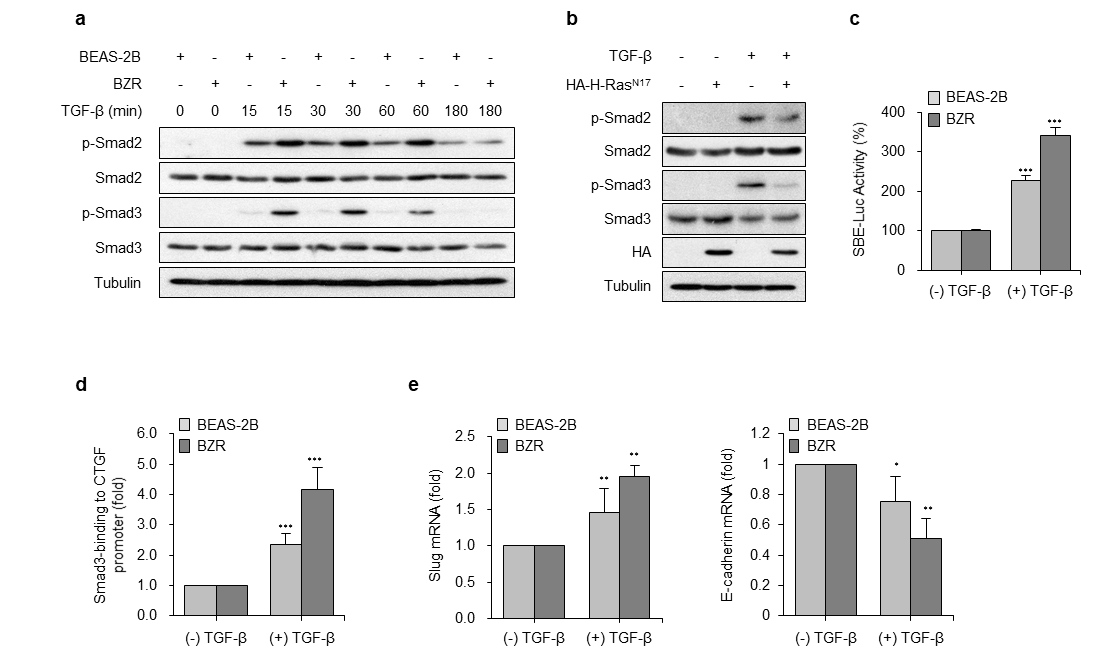
Supplementary figure 3. Ras promotes TGF-β-induced Smad2 and Smad3 activation.** (**a**) Phosphorylation of endogenously expressed smad2 and smad3 in BEAS-2B or BZR cells in response to TGF-β1 (5 ng/ml) for the indicated periods of time. (**b**) Expression level of p-smad2 and p-smad3 in BZR cells transfected with HA-tagged H-Ras^N17^ expression vector in response to TGF-β1 (5 ng/ml) for 1 h. (**c**) Luciferase activity of SBE-Luc reporter plasmid-expressed BEAS-2B or BZR cells in response to TGF-β1 (5 ng/ml) for 24 h. Columns, mean of three independent experiments performed in triplicate; *bar*, S.D.; ***P < 0.001 versus TGF-β1 untreated control. (**d**) ChIP assay to analyze smad3-binding to target promoters such as CTGF in BEAS-2B or BZR cells in response to TGF-β1 (5 ng/ml) for 1 h. Columns, mean of two independent experiments performed in triplicate; *bar*, S.D.; ***P < 0.001 versus TGF-β1 untreated control. (**e**) Real-time RT-PCR of slug (left panel) and E-cadherin (right panel) expression levels in BEAS-2B or BZR cells in response to TGF-β1 (5 ng/ml) for 24 h. Columns, mean of three independent experiments performed in triplicate; *bar*, S.D.; *P < 0.05, **P < 0.01 versus TGF-β1 untreated control.


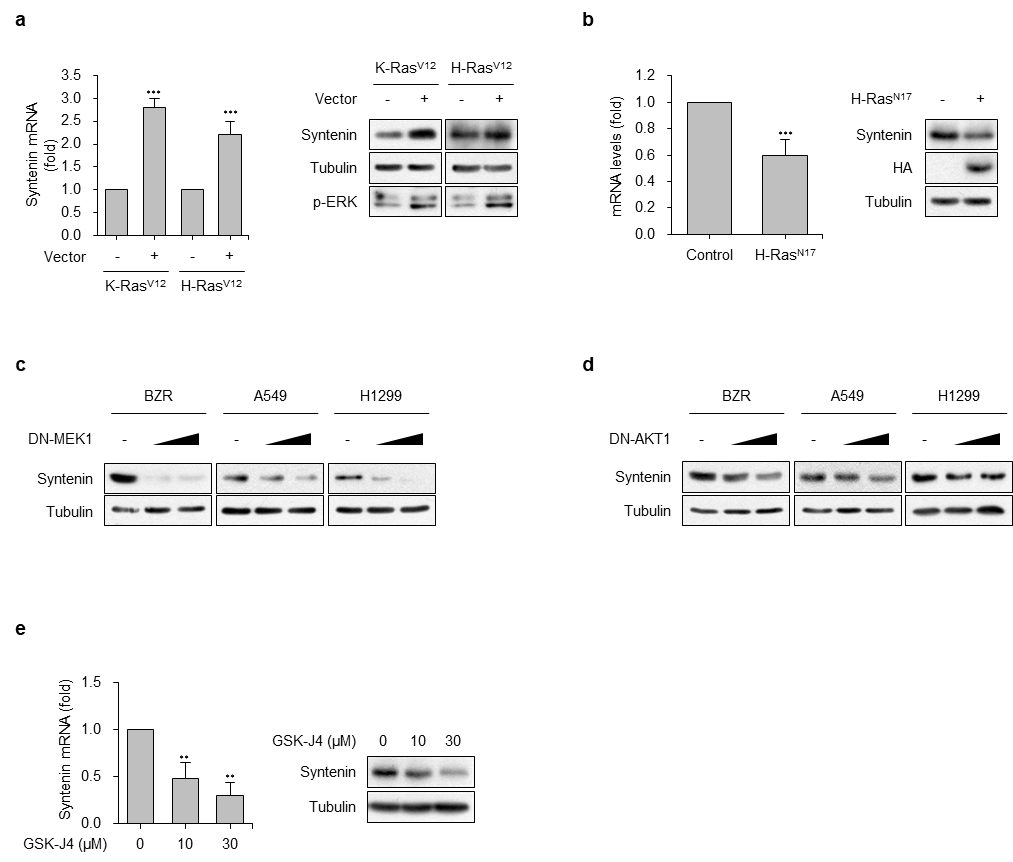


**Supplementary figure 4. Ras-JMJD3 axis regulates syntenin expression.** (**a**) Real-time RT-PCR (left panel) and western blot analysis (right panel) of syntenin levels in BEAS-2B cells transfected with K-Ras^V12^ or H-Ras^V12^ expression vector. Columns, mean of three independent experiments performed in triplicate; *bar*, S.D.; ***P < 0.001 versus control in each group. (**b**) Real-time RT-PCR (left panel) and western blot analysis (right panel) of syntenin levels in BZR cells transfected with H-Ras^N17^ expression vector. Columns, mean of three independent experiments performed in triplicate; *bar*, S.D.; ***P < 0.001 versus control. (**c**) Expression level of syntenin in BZR, A549 and H1299 cells transfected with the increasing amount of DN-MEK1 expression vector. (**d**) Expression level of syntenin in BZR, A549 and H1299 cells transfected with the increasing amount of DN-AKT1 expression vector. (**e**) Real-time RT-PCR (left panel) and western blot analysis (right panel) of syntenin levels in BZR cells in response to GSK-J4. Columns, mean of three independent experiments performed in triplicate; *bar*, S.D.; **P < 0.01 versus control.

**
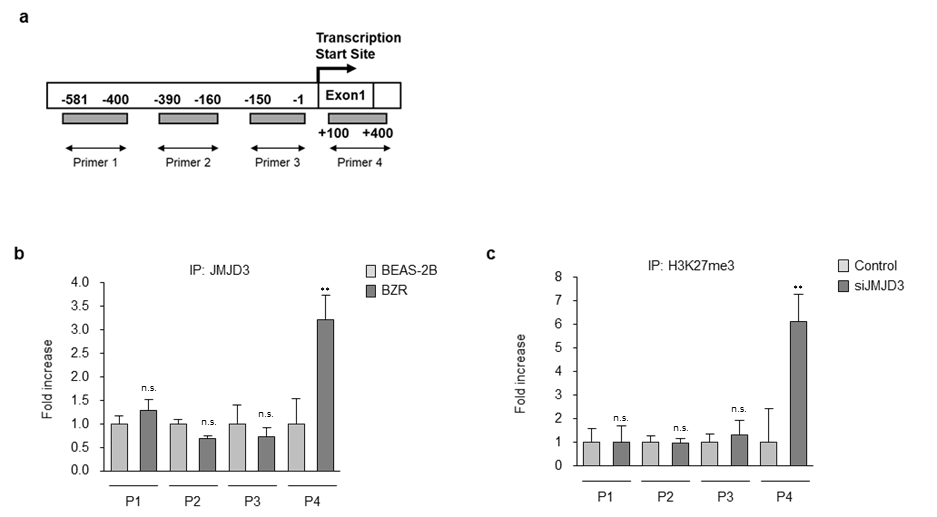
Supplementary figure 5. ChIP analysis of JMJD3 binding sites in the human syntenin promoter.** (**a**) Schematic representation of primer design for the ChIP assay in the human syntenin promoter region. Primers for ChIP assay are P1 (primer1), P2 (primer2), P3 (primer3) and P4 (primer4). (**b**) ChIP assay of JMJD3 recruitment at syntenin promoter using JMJD3 antibody in BEAS-2B or BZR cells. Precipitated DNA was amplified by Real-time qPCR with four primers flanking the predicted JMJD3 binding sites (shown) of the syntenin promoter. Columns, mean of two independent experiments performed in triplicate; *bar*, S.D.; **P < 0.01 versus BEAS-2B, n.s.: nonsignificant. (**c**) ChIP assay of H3K27me3 recruitment at syntenin promoter using H3K27me3 antibody in BZR cells transfected with control or JMJD3 siRNA. Precipitated DNA was amplified by Real-time qPCR with four primers flanking the predicted JMJD3 binding sites (shown) of the syntenin promoter. Columns, mean of two independent experiments performed in triplicate; *bar*, S.D.; **P < 0.01 versus control, n.s.: nonsignificant.

**
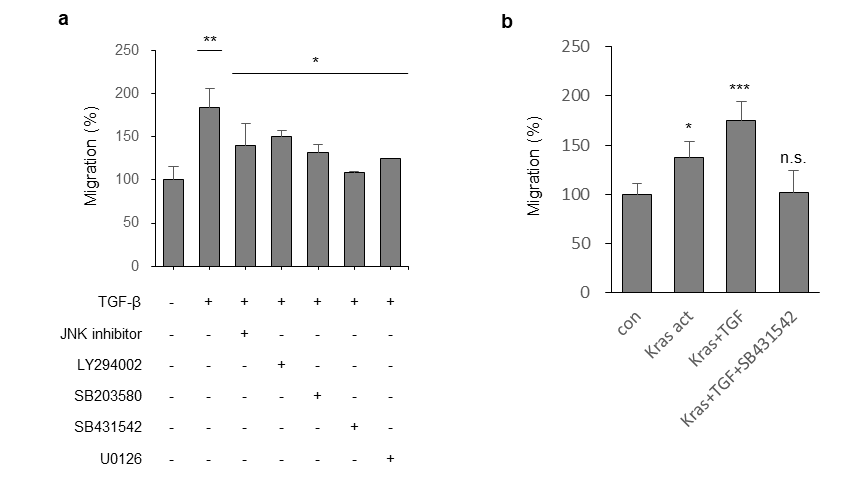
**

**Supplementary figure 6. Inhibition of TGF-β1 signaling pathway prevents Ras-promoted cancer cell migration.** (**a**) Transwell migration assay on BZR cells in response to with or without each inhibitors (10 *μ*M) ± TGF-*β*1, as indicated. Columns, mean of three independent experiments performed in triplicate; *bar*, S.D.; *P < 0.05, **P < 0.01 versus control. (**b**) Transwell migration assay on BEAS-2B cells in response to with or without SB431542 inhibitor ± Kras or TGF-*β*1, as indicated. Columns, mean of three independent experiments performed in triplicate; *bar*, S.D.; *P < 0.05, ***P < 0.001 versus control, n.s.: nonsignificant.

**
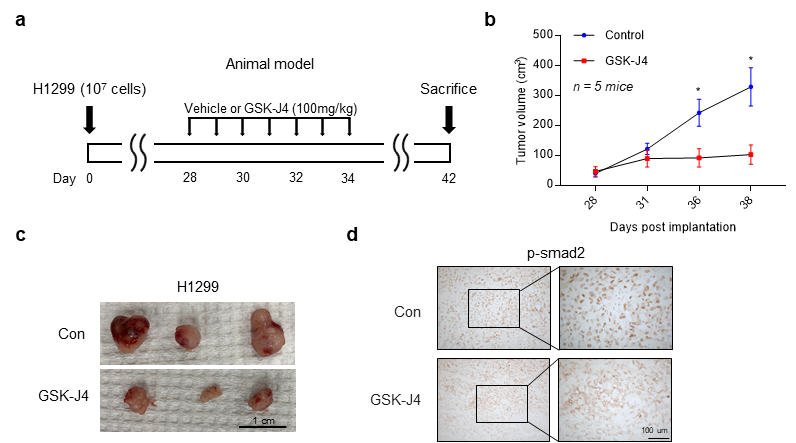
**

**Supplementary Figure 7. Repression of JMJD3 activity reduced tumor growth in mouse model.** (**a**) Schematic of experimental tumor xenograft model used. (**b**) Quantification volume of tumors generated from inoculation of H1299 at indicated days. The calculated average tumor volumes are presented are the means ± S.E.; *p < 0.05 versus control. (**c**) Gross appearance of primary tumors tissues in the tumor xenograft model in GSK-J4 groups and DMSO (control). Scale bar, 1 cm. (**d**) Representative images were stained with p-smad2 from primary tumor in each groups. Scale bar, 100 um.
