# Supplementary material for: Epigenetic regulation of TGF-β-induced EMT by JMJD3/KDM6B histone H3K27 demethylase
Source: Oncogenesis. 2021 Feb 26;10(2):17. doi: 10.1038/s41389-021-00307-0 (PMC7910473; doi:10.1038/s41389-021-00307-0)
Supplement: Supplementary file 2 — Epigenetic regulation of TGF-β-induced EMT by JMJD3/KDM6B histone H3K27 demethylase [file 41389_2021_307_MOESM2_ESM.docx]

**Table S1. Primary antibodies for Western blot analysis, immunohistochemistry, co-immunoprecipitation and immunofluorescence.**

| **Product name** | **Company** | **Catalog No.** |
| --- | --- | --- |
| p-ERK (Thr202/Tyr204) | Cell signaling | 9101s |
| p-Smad2 (Ser465/467) | Cell signaling | 3108S |
| Smad2 | Cell signaling | 3122s |
| p-Smad3 (Ser423/435) | Cell signaling | 9520S |
| Smad3 | Cell signaling | 9513S |
| Myc | Cell signaling | 2272S |
| goat anti-rabbit IgG | Cell signaling | 7074S |
| goat anti-mouse IgG | Cell signaling | 7076S |
| HA (hemagglutinin) | Cell signaling | 3724S |
| E-cadherin | BD Biosciences | 610182 |
| Vimentin | Santa Cruz Biotechnology | sc-6260 |
| TβRI | Santa Cruz Biotechnology | sc-402 |
| TβRII | Santa Cruz Biotechnology | sc-220 |
| Slug | Abcam | ab51772 |
| Histone H3 | Abcam | ab1791 |
| Histone H3 (Tri methyl K27) | Abcam | ab6002 |
| KDM6B/JMJD3 | Abcam | ab38113 |
| α-tubulin | Sigma-Aldrich | T5168 |
| Flag antibodies | Sigma-Aldrich | F3165 |
| Syntenin antibody | Abnova | H00006386-M01 |
| Anti-rabbit secondary Alexa 488 antibody | Molecular Probes (Invitrogen) | A-11008 |

**Table S2. Reagents for Western blot analysis, immunohistochemistry, co-immunoprecipitation and immunofluorescence.**

| **Product name** | **Company** | **Catalog No.** |
| --- | --- | --- |
| DAPI (4’,6-diamidino-2-phenylindole) | Sigma-Aldrich | D9542 |
| U0126 | Calbiochem | 662005 |
| LY294002 | Calbiochem | 440202 |
| GSK-J4 | Sigma-Aldrich | SML0701 |
| JIB-04 | Sigma-Aldrich | SML0808 |
| GSK-LSD1 | Sigma-Aldrich | SML1072 |
| Salirasib | Sigma-Aldrich | SML1166 |
| Recombinant human TGF-β1 | Millipore | GF111 |
| Protein A/G plus agarose beads | Santa Cruz Biotechnology | sc-2003 |

**Table S3. Primer sequences used in the study.**

|  | **Forward (5` 🡪 3`)** | **Reverse (5` 🡪 3`)** |
| --- | --- | --- |
| **E-cadherin** | CATCGCTTACACCATCCTCA | AGCTTGAACCACCAG GGTAT |
| **Slug** | CTGAGGATCTCTGGTTGTGG | CGAA CTGGACACACATACAG |
| **CTGF** | GCAGAGCCGCCTGTGCATGG | GGTATGTCTTCATGCTGG |
| **Smad7** | CCAACTGCAGACTGTCCAGA | TTCTCCTCCCAGTATGCCAC |
| **JMJD3** | GACCCTCGAAATCCCATCACAG | GTGCGAACTTCCACGGTGTGTT |
| **syntenin** | CACCATGACGATCCGTGACA | ATCAGTGAGG AGGCCGTTTC |
| **β-actin** | ACGTTGCTATCCA GGCTGTG | GAGGGCATAC CCCTCGTAGA |

**Table S4. Primer sequences used in ChIP analysis.**

|  | **Forward (5` 🡪 3`)** | **Reverse (5` 🡪 3`)** |
| --- | --- | --- |
| **CTGF** | ATATGAATCAGGAGTGGTGCGA | AACTCACACCGGATTGATCC |
| **Snail** | CGCTCCGTAAACACTGGATAA | GAAGCGAGGAAAGGGACAC |
| **Slug** | GCCTGCCTTTAGAGGGCTAC | TGCGCTACTCAGGGCTTC |
| **syntenin primer-1** | GGAATATTAGCTTAGGCGGACAGAGT | CCCTCTTGGGATGCCTTCGAC |
| **syntenin primer-2** | AACAGGGGCTCCCGAGACCT | GGGTAAAGAGGCCTGGGTGTG |
| **syntenin primer-3** | CTACCGCCCCGTTACCTTGG | GCAGTCTGTGAAGTGGCCC |
| **syntenin primer-4** | AGACTGCATCCTGGTCGC | AAACAAGGATCGAGAGACGGT |
